# Annotations

**Published:** 1892-03-19

**Authors:** 


### Annotations.

**Neurotic Novelists.**

M. Edmond de Goncourt evidently does not believe that a man is either a fool or a physician at forty. In his diary, just published, he lets his readers into many of the secrets of his life, and among other things he takes us into his confidence on the subject of his daily work. M. de Goncourt, at the present mature period of his age, apparently finds literary composition a somewhat difficult task. "Every day as I seat myself at my desk," he complains, "and say to myself, 'Now, then, you've got to drag another chapter out of your head,' I have the painful feeling of a man who should be asked day by day to part with some of his blood for the purposes of transfusion. . . . I have to work twelve hours, out of which only three are worth anything. At first a morning occupied by cigarettes . . . after lunch a long smoke . . . then till eleven at night correcting, cutting down, scratching out, and smoke drying with an infinite number of cigarettes." Now one does not like to call a man like M. de Goncourt by a harsh name, but no doctor who has anything like a true appreciation of his business can fail to feel that this French novelist deserves to be called by a very harsh name indeed. Can any man be really and truly sane who supposes that he is able to work at a literary occupation for twelve hours a day, and smoke cigarettes a considerable part of the time into the bargain? Novelists, more especially French ones, have the audacity to pride themselves upon their modernity. So far as the facts show anything to the contrary their modernity seems to consist in being entirely ignorant of everything that is modern, with the exception perhaps of the cut of modern coats and the quality of the newest slang. Of science, even of that physiological science which so profoundly concerns themselves and their working power, they appear to know absolutely nothing at all. They are mere outer barbarians and Central Africans. M. de Goncourt does not even seem to understand in physiology that two and three make five. He works twelve hours, he tells us, in order to get three hours' work done. But if he had the faintest glimmering of practical intelligence, he would spend six or seven of the hours that he now wastes in his study in some form of recreation or health-making employment. He would walk, or ride, or cycle, or dig in his garden, or play at rackets or rounders, or do all these things in turn, with a dozen others. Besides, he would by this time, if he had been as able as he is clever, have made himself acquainted with the cerebral and cardiac ruin brought about by the continuous smoking of tobacco. But in his behaviour towards these fundamentally important facts M. de Goncourt is like a peevish child. It is to be feared that he is by no means alone in his folly. Novelists and writers as a class pay small heed to physiologists and doctors. They pursue their own course for years, until their powers of brain, stomach, and heart are hopelessly impaired, and then they complain because a doctor cannot repair in a month the mischief which it has taken them twenty or thirty years to bring about. If novelists and writers generally want to be "modern" in a practically important sense they must take to the study of physiology; and what they learn in the course of their study they must practice with rational docility.

**The Bloodlessness of School Girls.**

The *British Medical Journal* quotes some important remarks by a well known German physician on the subject of the bloodlessness of girls at school. Dr. T. A. Reamy affirms that neglected cases of this class go from bad to worse, and finally die of tubercular consumption. This is an observation which every experienced physician can confirm from his personal knowledge. The whole question of the school life of girls is one eminently demanding investigation and the application of modern principles of hygiene. Reamy considers that the root of the difficulty lies in the fact that girls at school do not inhale sufficient oxygen: in other words they suffer, become delicate, and die from want of fresh air. By way of remedying this condition of things he proposes that there should be systematic deep inhalation for twenty minutes twice a day in a perfectly pure outside atmosphere. The deep inspiration should be carried out with the mouth closed, and under intelligent supervision. It is stated that no other known method of treatment more rapidly improves the character of the blood. Reamy, however, does not stop here; he insists that the patient shall leave school, give up all study, and spend several hours a day in the open air. Most judicious advice. He goes further, and orders abundance of beef steak and milk. For personal hygiene he prescribes the sponging of the body, including the extremities, every morning, with water of the temperature of the room, and afterwards friction with an ordinary towel. He does not despise blood-making medicine, and we should have very little faith in him if he did. Small doses of iron with a bitter tonic he recommends for continuous use. The question of the health of school girls is one of most serious importance. A deficiency of blood at the developing period of life impairs brain power, and makes school work so difficult as to be almost impossible. It makes growth stunted, renders the muscles feeble, impairs digestion, brings on constipation, and generally renders the period of girlhood and early womanhood a period of feebleness and misery. The consequences, even when death is escaped, are hardly ever repaired in after life. Many a melancholy mother of children owes her delicate health to the anæmia from which she suffered at school. The daily walk is an altogether inadequate substitute for the two or three hours vigorous romping and play which girls as well as boys need for their healthy development. Teachers of girls' schools, and all parents, would do well to make much more use of the services of intelligent medical men than they now seem disposed to do.

**The Miners' Holiday.**

The action of the coal miners, who this week make simultaneous holiday, will rouse in the mind of the average Briton, to whom this indulgence on the part of the purveyors of fuel is like to prove an expensive luxury, a wonder as to what manner of man these are, who can afford in such cavalier manner, to say when he will work and when he will play. Such liberty the middle-class citizen had believed to be the prerogative of dukes and other members of the richer classes. "I wish I could take a holiday when I please," he grumbles. So he could, if he chose to live as a miner does; if, having his present income, he lived in a house of one or two rooms, gave his children no education but what they were compelled to take gratuitously from the School Board, cared too little for his family to insure his life, and never by any chance expended a farthing in soap; nor had to pay income-tax, for though his income may exceed that of the hard-working clerk or struggling shopkeeper, from this burden your miner gets scot free. Yet he is not a rich man, as no man is rich who has not, at the day's end, a penny in his purse. And this is his case. Those who come into close contact with the collier — clergymen, doctors, teachers — tell marvellous but true stories of their improvidence. The present writer will give but two instances occurring in the same mining village. A man who was earning £7 a-week was accidentally shot by a drunken companion and disabled for some time. Within a fortnight of the accident he and his family were in receipt of parochial relief, being totally destitute. Another family, whose income averages £2 a-day—duly paid on Saturday—invariably have to ask for credit at the store on Tuesday morning. Nor are these isolated cases, but only examples of a common habit, and quoted because they can be personally vouched for by the writer, who lives in a district where these things occur, and where a publican can drive to church in a carriage and pair. The truth is, the miner, so long a slave, is still too largely animal. He does not recognise responsibilities, even of the domestic, still less of the national kind. His sole aim is to get the greatest possible amount of self-indulgence out of the largest attainable wage. We have done for him all that can be done by Act of Parliament; we have given him the franchise and the education code. But we have not yet made him a citizen.

